# Social, economic, and transboundary importance of foot and mouth disease quarantines in East Africa

**DOI:** 10.1016/j.jrurstud.2026.104256

**Published:** 2026-06-12

**Authors:** Ashley F. Railey, Susan D. Kerfua, Thomas L. Marsh

**Affiliations:** a Department of Sociology, Oklahoma State University, USA; b National Livestock Resources Research Institute, National Agricultural Research Organisation, Entebbe, Uganda; c School of Economic Sciences & Paul G Allen School for Global Health, Washington State University, USA

**Keywords:** Foot and mouth disease, Transboundary, Quarantines, Movement restrictions, Tanzania, Uganda, Governmentality

## Abstract

Although quarantines are vital for managing the spread of highly contagious livestock diseases, like foot and mouth disease (FMD) in East Africa, they can also create burdens for rural households whose livelihoods rely on the mobility and sale of their animals. In this paper, we used novel data from an outbreak of FMD to evaluate the importance of Ugandan livestock quarantines for 231 rural, livestock-owning households at the border of Uganda and Tanzania. Summary statistics and multivariate probit models evaluate the transboundary effects of quarantine restrictions on movement (human and international livestock movements, grazing) and sales (livestock products, live animals) and their relationship to household characteristics, country, and FMD virus infection. Results suggest that livestock movements and sales of live animals were important across both Tanzania and Uganda despite formal quarantine policies only in Uganda. FMD was strongly associated with quarantine restrictions, aligning with biosecurity goals, but households led by men and income diversity were also related to movements and grazing. Empirically, the results extend prior evidence from the study areas to show that herd infections shape the transboundary impact of public biosecurity practices but that differences between households can further affect quarantine response. Additional research is needed on connecting quarantine practices to specific changes in household biosecurity and exploring ways to minimize inequalities in disease response.

## 1. Introduction

The growth of the livestock sector in East Africa is hindered by infectious transboundary diseases that increase private and public disease control and prevention costs, reduce regional trade, and threaten food security (Baluka et al., 2014; Knight-Jones and Rushton, 2013). To manage biosecurity, authorities use quarantines, market closures, and movement restrictions on people and animals. This approach balances the risk of infection against the need to minimize severe, resource-intensive disruptions (Mumford, 2002). Examples worldwide suggest that disease events controlled with quarantine restrictions have direct effects on household livestock related activities (Limon et al., 2020). Particularly among rural, livestock-dependent populations where livestock disease has overlapping importance for household income and wealth (Marsh et al., 2016), human health (Subbiah et al., 2020), and nutrition (DeLay et al., 2020; Mosites et al., 2016), capturing the importance of quarantine restrictions to households can help define how the burden of disease is distributed.

In this paper, we use novel data from an outbreak of a highly contagious livestock disease, foot and mouth (FMD), in East Africa to assess the importance of quarantine restrictions for human and livestock movements, as well as sales of livestock and livestock products. FMD is a highly contagious, non-zoonotic virus that spreads rapidly among cloven-hoofed animals via direct contact, contaminated materials (fomites), and air. Because it easily transmits through livestock and environmental exposure, early detection and control are difficult (Knight-Jones and Rushton, 2013). FMD in East Africa faces further control challenges as an endemic disease (Kasanga et al., 2015; Kerfua et al., 2020). Endemic disease provides prior disease experience to facilitate identification of FMD symptoms and production impacts (e.g., lameness, reduced milk, and in extreme cases—mortality). Yet, the uncertain timing and magnitude of the virus within the herd deters households from changing livestock management practices before an outbreak (Railey et al., 2018). Frequent interactions between herds and limited vaccine supplies make prevention of FMD challenging for households (Ahmed et al., 2018), particularly during an outbreak (Muleme et al., 2012), in rural areas where veterinary services are already limited (Wolff et al., 2019), and across countries, which vary in their capacity for disease control (Allepuz et al., 2015; Baluka et al., 2014). Subsequently, public interventions tend to prioritize managing existing outbreaks over prevention, with methods differing by country.

Understanding the relationship between quarantine restrictions and FMD control in a transboundary, endemic setting aligns with efforts to improve the delivery of biosecurity (Gates et al., 2021) and may be particularly beneficial in East Africa where livestock contribute employment to over 70% of the population (Otte et al., 2012). Biosecurity efforts are often considered to reduce the economic impact to farmers and the sector (Tildesley et al., 2019) and stop disease spread (Bruckner, 2011). Yet, restrictions to movements of humans and animals, which are primary in quarantines, can have consequences for households beyond livestock income-generation (Kivaria, 2003; Limon et al., 2020; Rutagwenda, 2003). Indeed, pervasive differences in management of livestock by gender and access to diverse income sources are often associated with household impacts to economic growth, food consumption, and sustaining livelihoods (Alobo Loison, 2015; Tavenner et al., 2019; Tavenner and Crane, 2018). These same household characteristics likely shape the effects of biosecurity measures when household livelihoods center around livestock.

To that end, our approach seeks to examine the transboundary effects of quarantine restrictions across Uganda and Tanzania while evaluating how household socioeconomic characteristics and FMD infection relate to the importance of quarantines. We extend governmentality approaches that explain the relationship between disease and quarantines (Dean, 2009; Foucault, 1991) by considering the social and economic embeddedness of livestock households in East Africa (Granovetter, 1985; Polanyi, 1944). We combine bivariate statistics with multivariate probit analyses to test the transboundary importance of FMD governmentality for broader livestock-related movements (human and international livestock movements, grazing) and market-related sales (products, live animals) across both Tanzania and Uganda. The approach intends to better understand the burden of infectious, endemic disease in transboundary contexts with large livestock-owning populations and contribute to aligning public practices with varying disease impacts across households.

## 2. Background

### 2.1. Governing endemic disease

Biosecurity responds to microbial threats in a highly mobile social, economic, and environmental world by encompassing the regulatory activities that analyze, manage, and seek to reduce pathogen spread (Food and Agriculture Organization, 2007). A governmentality perspective (Dean, 2009; Foucault, 1991) is often adopted to explain biosecurity from the dual objectives of securing freedom from disease and ensuring the continuity of livestock-related activities (Hinchliffe and Ward, 2014). Individual and public freedom to avoid further disease impacts can involve unrestricted circulation of people and products, but the organization of security prioritizes disease prevention through ‘closure’ practices, such as through border controls, surveillance and monitoring, and controlled movements (Lentzos and Rose, 2009). That infectious disease spread imperfectly aligns with these technical means of spatial ‘closure,’ like quarantines, highlights the limits of disease governance (Enticott et al., 2012; Hinchliffe and Bingham, 2008; Mather and Marshall, 2011).

FMD in East Africa extends the spatial tension of governing infectious disease to include temporal tensions between knowing the likely impacts but having insufficient tools to prevent large-scale events (Bellet et al., 2021). Regional viral diversity (multiple virus types and sub-types) hinders large-scale, coordinated biosecurity efforts (Ahmed et al., 2018; Muleme et al., 2012; Railey and Marsh, 2019). Despite this complexity, recurring FMD outbreaks, documented at multiple intervals per year (Casey-Bryars et al., 2018; Kerfua et al., 2020), mean that households are well-acquainted with the disease. This endemicity ensures that households anticipate periodic infections and the resulting livestock and livelihood consequences. Livestock-owning households in East Africa report reduced milk production, weight loss, and reduced livestock grazing attributed to FMD (Baluka et al., 2014; Rutagwenda, 2003). The extensive consequences over time and space of endemic disease then shift the focus from disease prevention, to control and minimization of adverse effects (Bellet et al., 2021). The temporal certainty of an endemic disease with clear impacts also serves to distinguish it from the more uncontrollable exotic diseases that are low in probability and incalculable in impact (Beck, 1996). As a result, rural households in East Africa report limited uptake of preventative measures, mainly focusing on limiting impacts once herds become infected (Chenais et al., 2019; Mutua and Dione, 2021; Wolff et al., 2017).

Quarantines are one technical approach to address freedom from disease and can be viewed as part of a larger governmentality program to control disease risk (Dean, 2009:218). Both Tanzania and Uganda rely on routine national surveillance during disease outbreaks (Kerfua et al., 2020). FMD is considered a notifiable disease of public concern where the disease is to be reported to the relevant authorities within the shortest possible time after detection (MAAIF, 2023; Uganda, 2000). This then sets off actions for testing animals. Reports from Tanzania suggest that subsequent restrictions on movements and quarantines are often not enforced or implemented effectively due to farm-, community/district-, and national-level barriers, including limited personnel, lack of reporting or communication, and diverging individual priorities (Ndhlovu, 2016). Uganda differs from Tanzania by enforcing farm-level biosecurity through emergency vaccination (Muleme et al., 2012) and establishing formal quarantine areas (Uganda, 2000:7). Quarantine restrictions can last beyond a year in some cases and are deployed at sub-national (district) levels (Muleme et al., 2012). The decentralized management of veterinary services within Uganda means that households, local leaders, and potentially government veterinarians may not fully appreciate the importance of abiding by quarantines (Ilukor et al., 2015; Wolff et al., 2019). Across both countries, livestock owning households are predominantly rural, which can further limit timely access to and the availability of biosecurity measures (Wolff et al., 2019). Similar to other cases of endemic FMD where disease efforts are stratified across countries (Rich and Winter-Nelson, 2007), the dispersion of responsibility for upholding quarantines across individual households, government and non-government veterinarians, as well as political boundaries may limit biosecurity while extending the impacts of disease across both sides of the Uganda-Tanzania border (Allepuz et al., 2015; Baluka et al., 2014).^1^

### 2.2. Household embeddedness

Where the governmentality perspective helps explain the relationship between disease and biosecurity practices, the embeddedness perspective helps define how households navigate a range of impacts when livelihoods carry social and economic importance (Granovetter, 1985; Polanyi, 1944). Indeed, Foucault and others emphasize the populace as sources of critical challenge to public interventions (Foucault, 1991; Li, 2007; Rose, 2001). For rural households in East Africa, livestock are primary to ensuring livelihoods (Otte et al., 2012) and have been linked to reducing perpetual poverty (Grace et al., 2017) and associated with human welfare (DeLay et al., 2020; Marsh et al., 2016). Yet, significant variation across households exists on accessing strategies to manage livestock (Alobo Loison, 2015; Poortinga et al., 2004; Quisumbing et al., 2014).

Income from livestock activities is a primary measure of engagement in the sector but, given the limited opportunities for preventing FMD in East Africa, may be insufficient to explain why quarantine restrictions are important. For example, evidence from rural-livestock owning populations suggest that diverse income portfolios provide alternative sources of cash flow (Alobo Loison, 2019), which can help smooth consumption during reduced productivity (Tesfaye and Tirivayi, 2020), such as when livestock markets are closed during quarantines. For FMD, the impact of changes to market prices for livestock and livestock products may partially be addressed by engaging in agricultural or off-farm income generation, which can be protective during an outbreak (Kerfua et al., 2023). More broadly, during FMD outbreaks globally, households and communities with limited livelihood diversification face increased risk of adverse economic consequences that could be tied back to both infection and larger public response (Limon et al., 2020; Poortinga et al., 2004).

In East Africa, the head of household gender provides an additional indicator on access to opportunities to engage in livestock markets. Women traditionally comprise a significant portion of rural livestock ownership in East Africa, but gendered relations introduce socioculturally differentiated management of household activities and engagement in the livestock sector (Kristjanson et al., 2010; Quisumbing et al., 2014). For example, a gendered evaluation of dairy production in Kenya found that livestock activities were predominantly viewed as masculine, which privileged men in the production of livestock and the associated benefits (Tavenner and Crane, 2018), such as access and control of information. Pervasive gender norms among East African livestock owning populations have constrained women's participation in community affairs and their access to productive resources such as land and capital (Dolan, 2004; Rietveld, van der Burg, and Groot, 2020). For FMD, evidence of disparities in head of household gender are reflected in interpretations of vaccine quality whereby men are willing to pay more for vaccines but less for vaccines of lower efficacy compared to women (Railey et al., 2018). Similar disparities exist in understandings of risk (Doss et al., 2008; Hannah-Moffat and O'Malley, 2007), which may signal gendered differences in acquiring information or access to resources (Torkelsson and Tassew, 2008).

Finally, the embeddedness perspective extends governmentality's views on the tension between disease spread and the limits of public biosecurity by positioning the transboundary distribution of quarantine impacts as resulting from the structure of networked relations (Granovetter, 1985; Polanyi, 1944). Uganda and Tanzania are part of a primary East African cattle trading route, which extends from Tanzania throughout Uganda into Kenya and South Sudan. The Cattle Corridor is characterized by spatially distant outbreaks of FMD as well as highly interconnected livestock movements (Hasahya et al., 2023). Yet, the livestock sector in East Africa is marked by limited integration across the value chain between farmers, beef processors, and traders (Mpairwe et al., 2015; Waiswa et al., 2021), which encourages informal and formal movements across borders and to livestock markets (Ekwem et al., 2023; González-Gordon et al., 2023). The diverse and diffuse relations within the livestock sector then likely facilitate movements and sales during quarantines, regardless of the ‘absence’ or ‘presence’ of the FMD virus or whether a household is in Uganda or Tanzania.

### 2.3. Current study

We combine the considerations offered by the governmentality and embeddedness perspectives alongside biosecurity practices in East Africa to define which quarantine restrictions are important for which households. First, formal quarantine measures in Uganda on the border of Tanzania suggests evaluating the importance of quarantines for households across both countries. The importance of quarantine restrictions for households in East Africa is likely partially accounted for by detection of FMD in the herd and household socioeconomic characteristics. Specifically, the spatial and temporal importance of endemic FMD is likely related to a range of quarantine consequences. Household income diversity and head of household gender as indicators of socioeconomic embeddedness in the livestock sector likewise should relate to the importance of multiple quarantine impacts. Indeed, the embeddedness perspective further indicates that households across both Uganda and Tanzania are linked by their livelihood strategies, which make quarantines important across a range of restrictions. This last point also aligns with the challenge of achieving stratified approaches to disease control across countries and tensions with disease spread.

## 3. Materials and methods

### 3.1. Study design

Data collection occurred in 2018 in the Kyaka and Nsunga wards in Tanzania and the Ugandan sub-counties of Endinzi, Lwamaggwa, and Kakuuto. The study sites were chosen based on their location at the center of informal and formal livestock activities along the Cattle Corridor and international border between Tanzania and Uganda. The study sites primarily comprise rural areas, with some central towns (population density ranged from ~72 to 257 people per square kilometer).^2^ Households were randomly selected from within sub counties and wards where an FMD outbreak had previously been verified within the past year (Kerfua et al., 2018) and based on a list of livestock owners provided by the District Veterinary Officers. At the time of data collection, formal quarantines had been implemented across the study sites in Uganda at some point in the last year. A two-stage sampling process selected households, first by district then households within the district, with Uganda more intensively sampled to facilitate analysis across household livestock management practices (e.g., live animals, agriculture, off-farm income) (Kerfua et al., 2023). Households provided recall data on household and quarantine behaviors from the past year. Households were compensated for their participation. Relying on local veterinarians and community leaders to identify and contact households within a multi-day data collection window helped reach households. As a result, of those sampled (170 households from Uganda and 85 households from Tanzania), 256 completed the survey (97% response rate). Additional missing data appeared seemingly at random, with missing variables occurring in no more than 10% of the variables included in the analysis. The final sample size reflected those who provided responses to the questions used in the analysis (n = 231).

### 3.2. Dependent variables

Five different quarantine restrictions are evaluated, including for movement (human and international livestock movements, grazing) and sales (livestock products, live animals). The restrictions capture the primary aims of quarantines in the region,^3^ in response to FMD detection in the area, and intend to capture effects beyond direct market impacts. Households were asked ‘how would you rank the effect of quarantine restrictions on your household?’ using the scale ‘not important,’ ‘somewhat important,’ or ‘very important.’ We dichotomize these impacts to reflect ‘not important’ (=0) compared to ‘somewhat/very important’ (=1).

### 3.3. Covariates

FMD infection within the household herd in the past 12 months captures the presence of disease. Household's reported FMD as either ‘yes’ for FMD positive results (=1) or ‘no’ for FMD negative results (=0). One household that responded with ‘I don't know’ was excluded from the analysis. Country differences in biosecurity measures categorize household location as either Uganda (=1) or Tanzania (=0). The primary household characteristics of interest include socioeconomic considerations for the head of household gender (man = 1, woman = 0) and income diversity (agricultural and off-farm income). Income diversity is based on whether the household primarily reported income from off-farm and other agricultural activities (non-livestock) (=1), compared to those who also reported income from livestock activities (=0).

Additional variables include herd size (number of cattle, goats, and sheep owned) and the highest level of education completed by the head of household (no formal school = 0, primary/secondary/tertiary or University and higher = 1). Apart from herd size, the household socioeconomic characteristics enter as categorical data. Herd size is log-transformed such that the effect of herd size on livestock inputs increases at a diminishing rate as herd size grows.

### 3.4. Analytical strategy

Our analysis examines both the importance of quarantine restrictions across countries and the relationship between quarantine restrictions with household socioeconomic characteristics and disease. We start with bivariate comparisons of the importance of quarantine restrictions for movements (human and international livestock movements, grazing) and sales (livestock products, live animals) by country. We next evaluate how households view the importance of quarantine restrictions by employing a multivariate probit model to simultaneously estimate the relationship between the household socioeconomic characteristics and disease on each of the quarantine restrictions while accounting for the relationship between the restrictions.

The multivariate probit approach addresses the interrelatedness of household livelihood decisions, which are likely linked by unobserved factors such as at the market-level (price changes, informal trade) and household-level (tradeoffs in consumption and production). For example, restrictions on milk sales may result in increased consumption of milk within the household (Baluka et al., 2014; Kerfua et al., 2023). The correlation in the error term across the restriction models then arises from these unobserved factors. Separate models by quarantine restriction overlook the correlation in error terms, which can lead to bias and inefficient coefficient estimates (Greene, 2003). Formally, a system of equations captures the expected importance of quarantine restrictions for households as a linear function of the observed household (*i*) disease and socioeconomic characteristics (*X*) with multivariate normally distributed stochastic terms (*ϵ*_*ij*_):

$$Y_{ij}^{*}=X_{ij}\beta_{j}+\epsilon_{ij},\;\text{where}\;j=1,2,3,4,5$$

Where $Y_{ij}^{*}$ is the unobserved expected importance of restriction *j* for household *i* and *X*_*ij*_ is a vector of variables which are hypothesized to influence the importance by the error term *ϵ*_*ij*_, which is correlated across *j*. The household importance of each quarantine restriction is described by the observable binary choice:

$$Y_{ij}=\left\{\begin{array}{c} \begin{array}{cc} 1 & if\;Y_{ij}^{*}>0 \end{array}\\ \begin{array}{cc} 0 & \text{otherwise} \end{array} \end{array}\right.$$

The multivariate normal distribution of the error term with a conditional mean of zero and variance equal to one, accounts for the relationship between quarantine restrictions through the symmetric variance-covariance matrix Σ, given by:

$$\Sigma =\left[\begin{array}{cccc} \begin{array}{c} 1\\ \rho_{21}\\ \vdots \\ \rho_{j1} \end{array} & \begin{array}{c} \rho_{12}\\ 1\\ \vdots \\ \rho_{j2} \end{array} & \begin{array}{c} \cdots \\ \cdots \\ \ddots \\ \cdots \end{array} & \begin{array}{c} \rho_{1k}\\ \rho_{2k}\\ \vdots \\ 1 \end{array} \end{array}\right]$$

The *ρ*_*jk*_ represents the pairwise correlation coefficient of the error terms associated with any two quarantine restrictions *j* and *k* estimated by the model. When the error term correlation *ρ*_*jk*_ is present, the off-diagonal elements become non-zero. Thus, shared unobserved factors, such as market access or household tradeoffs in livelihood strategies, which can affect the importance of quarantines across a range of restrictions, are captured by *ρ*_*jk*_. Strong correlations between the restrictions can be interpreted as > 0.50 and support the widespread importance of quarantines as related to embedded socioeconomic processes.

The results are reported as average marginal effects to facilitate comparison across models. Average marginal effects for continuous variables are interpreted as percentage-point changes in the dependent variable associated with a one-unit increase in the predictor, while binary or categorical variables show the discrete change between predictor levels. Sensitivity analyses evaluate separate probit models to test the joint multivariate probit model specification and how many quarantine restrictions households reported as important, using a Poisson model. Across all analyses, standard errors, and log-likelihoods inform model fit, with log-likelihood tests comparing across models.

## 4. Results

Table 1 illustrates the summary statistics of the sample (n = 231). Following virological sampling in the area, the reported prevalence of FMD is high with 71% of the total sample reporting FMD in the herd within the past year. Similar to existing evidence on livestock ownership in East Africa, most households report men as heads of households (91%). Households report receiving income from a combination of agriculture and livestock activities (52%), with the fewest households reporting off-farm income and agriculture as their primary income source (14%).

**Fig. 1.**
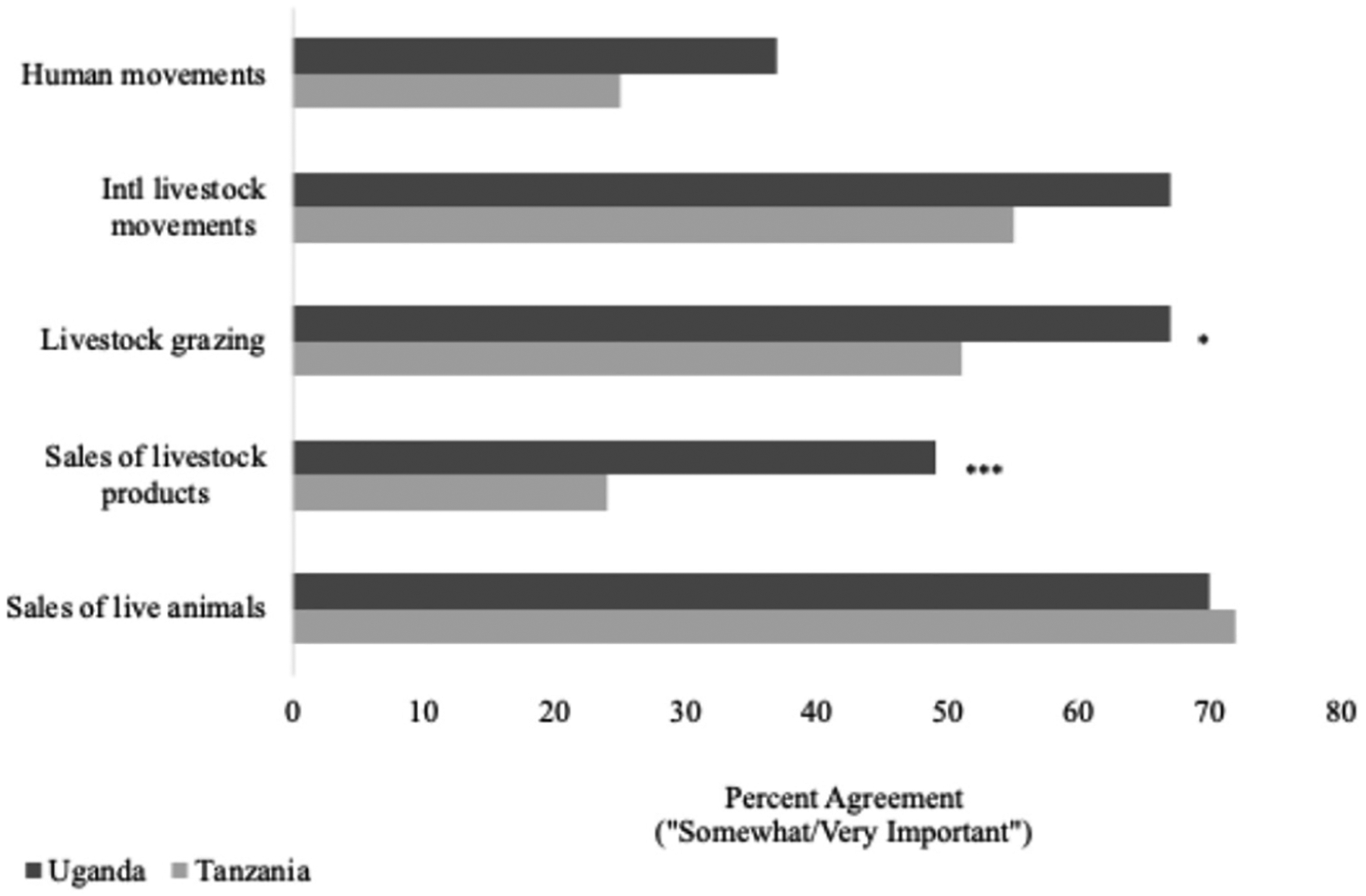
Importance of quarantine restrictions by country (n = 231) Note: Pearson chi-square test for categorical variables. P value: *p < 0.05, **p < 0.01, ***p < 0.001.

Among the quarantine restrictions (Fig. 1), the most important restrictions across both countries are livestock sales, grazing, and international livestock movements. The importance of livestock grazing and sales of livestock products differ by country (p < 0.05), with households in Uganda ascribing more importance to the effect of these restrictions (grazing: 67% vs. 51%; livestock products: 49% vs 27%, in Uganda and Tanzania respectively). The full distribution of the restrictions by country reflects these findings and appears in the appendix (Fig. A1).

The relationship between the importance of quarantine restrictions with FMD and household socioeconomic characteristics appears in Table 2. The multivariate probit model fits the data, rejecting the null hypothesis that the covariance of the error terms across equations are equal to zero (likelihood ratio test *χ*^2^ (10) = 160, p < 0.001). This supports the use of the multivariate probit over the separate probit models (Appendix Table A1) and the joint importance of quarantine restrictions. FMD infection in the herd appears to drive the importance of quarantine restrictions for market-related activities. Households with FMD positive results have a higher probability of reporting sales of livestock products (21%-points) and livestock (27%-points) as important restrictions compared to FMD negative results. FMD is also related to human movements (20%-points). Compared to households who primarily rely on livestock and agriculture for income, households who also receive off-farm income have a lower probability of reporting human movements as important (–27%-points). The effects across income and FMD align with viewing quarantine restrictions as directly related to livestock activities but also likely related to maintaining livestock as part of household livelihood strategies (broader human and livestock movements). Households led by men are additionally less likely to report quarantine restrictions for international movements (20%-points) and livestock grazing as important (25%-point) compared to woman-led households. This likely relates to household approaches to shifts across interrelated markets and livelihoods.

The analysis of the model's correlation matrix further provides insights into the overlap of quarantine impacts (Table 3). Positive, strong (>0.50), and statistically significant correlations indicate high interrelatedness or complementarity, where ranking one restriction as important increases the probability of ranking the other as important. Restrictions to human movements, international movements, and grazing are strongly related, while grazing restrictions are additionally related to livestock sales. The strong interdependence of restrictions supports household embeddedness in livestock activities. Specifically, these restrictions appear related to the primary activities associated with maintaining livestock for income-generation, which may be driven by market changes or intrahousehold decisions that link a range of livelihood strategies.

### 4.1. Additional results

**Fig. 2.**
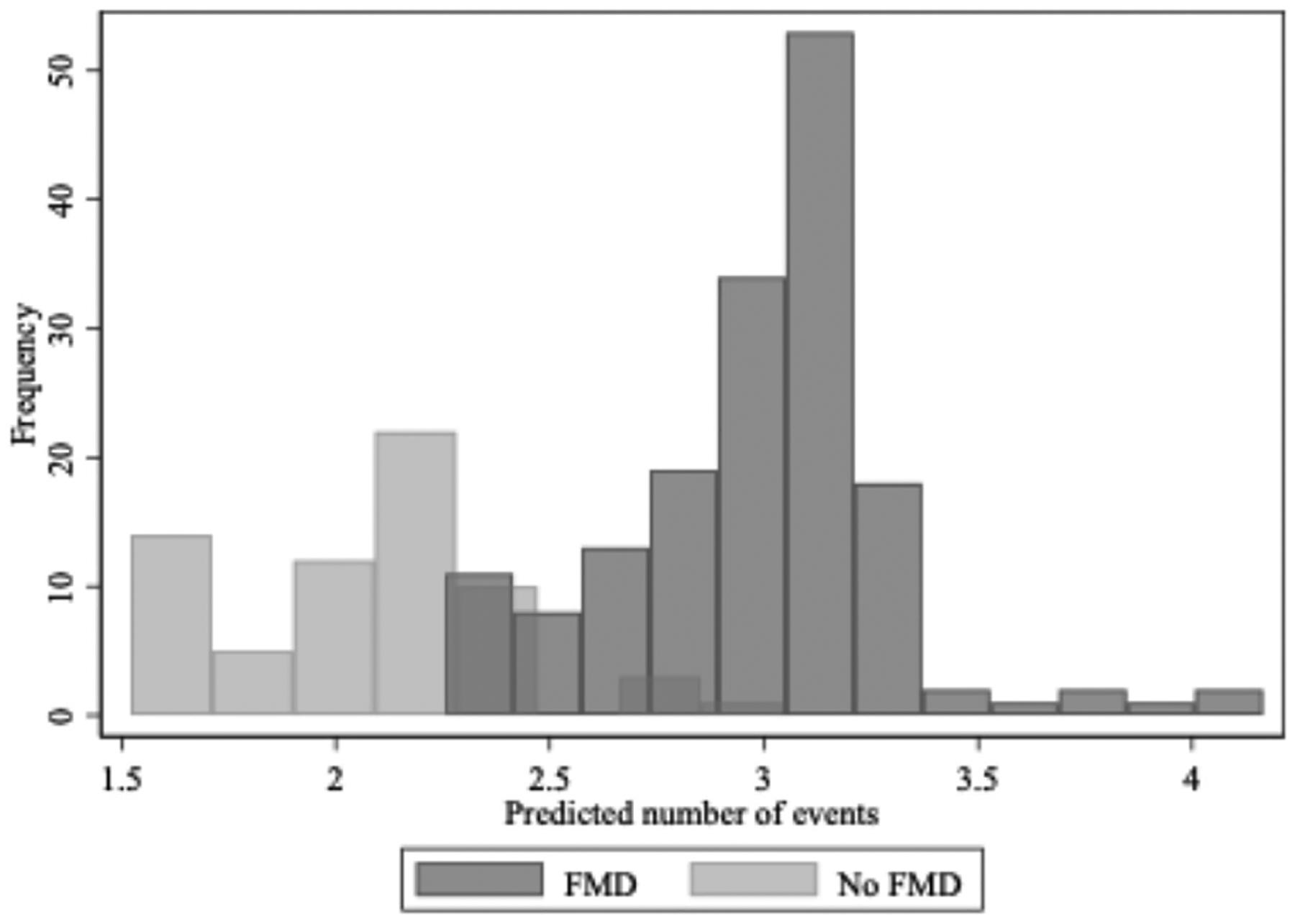
Predicted number of important quarantine restrictions by FMD (n = 231) Note: Predicted count from Poisson model (Appendix Table A2).

We additionally assess the number of quarantine restrictions ranked as important to define the intensity of quarantine restrictions. First, 89% of households report that at least one restriction is important with most households reporting three restrictions (24%, range 0–5). FMD primarily relates to the predicted number of restrictions reported (Fig. 2). Households with FMD positive results are predicted to report three restrictions while those with FMD negative results are predicted to report two restrictions. Men similarly are predicted to report one less restriction compared to women-led households (three versus four restrictions, respectively). These findings strongly support households with FMD as experiencing the greatest burden from quarantines, as well as providing some additional evidence on gender differences in managing the burden of disease.

## 5. Discussion

Our study evaluates the transboundary importance of FMD quarantine restrictions across Tanzania and Uganda to advance our understanding on how disease and households shape governance practices to address the burden of endemic disease. Aligning with our theoretical perspectives, households report significant restrictions attributed to quarantines, regardless of country and infection status, which emphasizes the tension between disease spread and biosecurity efforts at the Uganda-Tanzania border (Allepuz et al., 2015; Baluka et al., 2014), reflects extant empirical evidence on widespread quarantine impacts (Baluka et al., 2014; Limon et al., 2020), and supports livestock activities as highly embedded across diffuse socioeconomic activities (Otte et al., 2012). Notably, over 60 percent of households across both Tanzania and Uganda reported that quarantine policies—which were enforced only in Uganda—had important effects on livestock sales, grazing, and international movements. Herd infection status and household socioeconomic characteristics help further explain the importance of these restrictions.

First, following insights into the governmentality of infectious livestock and extending considerations for chronic animal disease (Bellet et al., 2021; Holloway et al., 2024), the spatial and temporal complexity of controlling FMD requires coordination of private and public approaches. Specifically, the strong and consistent relationship between FMD and restrictions on livestock and product sales aligns with prior findings in the study regions (Kerfua et al., 2023) and the role of FMD in livestock productivity. Disease and quarantines then overlap in addressing opportunities for market access and overall continuity of business during an outbreak, which extend across countries and households. One option to further align quarantine restrictions with disease incidence is through targeted vaccination. Vaccination during outbreaks has shown some feasibility in Uganda (Muleme et al., 2012) with households in Tanzania willing to pay for vaccines in response to spatial and temporal proximity (Railey et al., 2018). The next step would consider the benefit of vaccination during quarantines, such as allowing movements with proof of vaccination, which aligns with concerns for transboundary movements and elevated impacts for households with FMD. Proactive FMD vaccination in contrast has shown some feasibility in East Africa (Casey-Bryars et al., 2018), but would require similar considerations for coordination across countries, quarantine areas, and households, as well as equitable allocation and appropriate funding (Ilukor et al., 2015). Indeed, prior subsidization of vaccines in Uganda may affect households’ willingness to pay and perceived responsibility for vaccination (Wolff et al., 2019). Moreover, for those households experiencing FMD, disease severity, such as for morbidity and mortality, can shape household preferences for when disease (and subsequent public interventions) are needed (Holloway et al., 2024). Providing households with the skills and knowledge of biosecurity to then act upon these preferences is needed in East Africa (Wolff et al., 2019). Local, community approaches that link public practices with household expertise show promise in overcoming gaps in biosecurity (Catley and Leyland, 2001).

The social and economic importance of livestock in East Africa reinforces diverging effects of quarantines across households. Head of household gender and income diversity are primarily associated with the importance of quarantine restrictions for grazing and livestock movements. In part, the small sample size of woman-led households across both countries is an indication of the position of women in the livestock sector but the results further support that where these differences are most evident are in access to resources within the livestock sector. For example, men are predicted to report one less quarantine restriction as important than women. The overlapping importance of livestock for social and economic benefits would suggest that the reduced impact of quarantines is rooted in social norms that can make productive participation in markets challenging (Tavenner and Crane, 2018) or stratified along gender-lines to prevent access to important information (such as grazing or alternative markets) (Torkelsson, 2007). Directing resources and tailored information to women's networks may help address the gap in impact, particularly through empowerment and decision-making during a time when household options are limited (Madzorera et al., 2023; Njiru et al., 2024). Similarly, where households led by men or with diverse income sources can self-insure against movement restrictions, alternative grazing options, such as providing feed, and public investments in non-farm assets (education, land resources, farm inputs) may abate the unequal impact of quarantines on households. Importantly, these inequalities likely interact and reinforce each other (Vincent, 2022), especially as women-led households in rural East Africa face greater time poverty related to accessing these assets (Bain, Ransom, and Halimatusa'diyah 2018).

Our paper has several limitations. First, the retrospective data collection introduced the potential for recall error and social desirability bias that may influence the accuracy of the estimates. However, the familiarity with FMD and quarantine restrictions suggests our approach may broadly capture the most salient effects of quarantines during an FMD outbreak. Additional analyses should also look to connect household reports on the importance of quarantine restrictions to changes in markets and household livestock practices. Similarly, the conceptualization of household opportunities to access resources reflects proxies for more precise social embeddedness processes. Further investigation into the specific forms of embeddedness, such as through social capital, or changes in livelihood diversification, would enhance our discussions. Our measure of income would likewise benefit from more precise measurement, as the current measurement is likely biased due to endogeneity by using livestock income as a comparison category. This would help explain the limited effect on live animals and livestock product sales. As is, our study provides initial findings on the contribution of quarantine policies to the distribution of the burden of disease.

## 6. Conclusions

Quarantine restrictions during an FMD outbreak in East Africa had widespread, transboundary effects across household livestock activities with disproportionate impacts on households with FMD infected herds and by household socioeconomic characteristics. The findings align with increasing evidence on the social and economic importance of endemic disease while also signaling the need for evaluating how households differentially experience disease and biosecurity practices. Further research is needed on connecting quarantine restrictions to household biosecurity practices and exploring ways to minimize inequalities in disease response.

## Supplementary Material

Supplementary Material

## Figures and Tables

**Table 1:** Summary statistics (n = 231).

	n (%)
Herd Size, m (sd)	54 (76)
Highest level of Education (=Primary or higher school)	76 (33)
Primary Income Source (=Agriculture & off-farm income)	33 (14)
Head of Household Gender (=Man)	210 (91)
FMD Herd Status	
FMD Herd Status (Yes, infection)	164 (71)

Notes: Herd size includes cattle, sheep, and goats. Reference categories: Education = no formal education. Primary Income Source = Livestock only or livestock and crops. Gender = Woman. FMD = No reported FMD in past year. N = number; % = percent; m = mean; st = standard deviation; FMD = foot-and-mouth disease.

**Table 2:** Relationship between household and FMD characteristics with the importance of quarantine restrictions (n = 231).

	Human movements		International livestock movements		Livestock grazing		Sale of livestock products		Sale of livestock	
	AME	SE	AME	SE	AME	SE	AME	SE	AME	SE
Herd size	−0.01	(0.67)	0.04	(0.23)	0.01	(0.68)	0.05	(0.07)	0.04	(0.15)
Head of Household Education Primary or higher school	0.07	(0.33)	−0.01	(0.92)	−0.00	(0.99)	0.04	(0.54)	−0.08	(0.21)
Head of Household Gender Man	−0.07	(0.54)	−0.20*	(0.03)	−0.25**	(0.00)	−0.15	(0.18)	−0.12	(0.15)
Primary Income Source Agriculture & off-farm	−0.27***	(0.00)	0.06	(0.54)	0.17	(0.05)	−0.08	(0.42)	0.09	(0.28)
FMD Herd Status Yes, infection	0.20***	(0.00)	0.05	(0.50)	0.13	(0.08)	0.21**	(0.00)	0.28***	(0.00)
Country Uganda	0.05	(0.54)	0.08	(0.33)	0.20*	(0.02)	0.14	(0.09)	−0.08	(0.26)
Log-Likelihood	−629									

Note: Multivariate probit regression models, with standard errors in parentheses and 45 degrees of freedom. Quarantine impacts; 1 = Somewhat/very important, 0 = Not important. Reference categories: Highest level of education completed = No formal education. Primary Income Source = Livestock only or livestock and crops. Gender = Woman. FMD herd status = No reported FMD in past year. Country = Tanzania. AME = average marginal effects. SE=standard error. FMD= Foot and mouth disease. *χ*^2^ (10) = 160, p < 0.001 where null hypothesis is covariance of error terms are not correlated.

P value:

* p < 0.05,

** p < 0.01,

*** p < 0.001.

**Table 3:** Correlation matrix of important quarantine restrictions (n = 231).

	Human	International	Grazing	Products	Livestock
Human	1				
International	0.76***	1			
Grazing	0.54***	0.67***	1		
Products	0.12	0.24*	0.13	1	
Livestock	0.11	0.38***	0.61***	0.41***	1

Note: Correlations from multivariate probit analysis.

P value:

* p < 0.05,

** p < 0.01,

*** p < 0.001.

## Data Availability

Data will be made available on request.

## Footnotes

1 Kahungu, MT (2 February 2024). Government outlines foot and mouth disease fighting plans as MPs call for compensation. *Parliament Watch*. https://parliamentwatch.ug/news-amp-updates/government-outlines-foot-and-mouth-disease-fighting-plans-as-mps-call-for-compensation/.

2 Rural areas consist mostly of low-density population (<300 people per square kilometer). (10 March 2020). https://blogs.worldbank.org/en/sustainablecities/how-do-we-define-cities-towns-and-rural-areas#:~:text=Towns%20and%20semi%2Ddense%20areas%2C%20which%20have%20a%20population%20of,300%20inhabitants%20per%20km2.

3 https://ugandatrades.go.ug/media/136_1Animal_Diseases_Rules_(S.I._38_4).pdf.

## Appendix A. Supplementary data

Supplementary data to this article can be found online at https://doi.org/10.1016/j.jrurstud.2026.104256.

## Glossary:

N: number
%: percent
m: mean
st: standard deviation
FMD: foot-and-mouth disease
