# Supplementary material for: Social, economic, and transboundary importance of foot and mouth disease quarantines in East Africa
Source: J Rural Stud. Author manuscript; Available in PMC 2026 Jul 29. (PMC13384485; doi:10.1016/j.jrurstud.2026.104256)
Supplement: Supplementary Material [file NIHMS2194301-supplement-Supplementary_Material.docx]

**Appendix**

**Figure A1** Distribution of the importance of quarantine restrictions by country (n=231)

**Table A1** Household socioeconomic characteristics and FMD incidence on important quarantine restrictions (n=231)

|  | Human movements | | International livestock movements | | Livestock grazing | | Sale of livestock products | | Sale of livestock | |
| --- | --- | --- | --- | --- | --- | --- | --- | --- | --- | --- |
|  | AME | SE | AME | SE | AME | SE | AME | SE | AME | SE |
| Herd size | 0.02 | (0.47) | 0.03 | (0.26) | -0.01 | (0.79) | 0.06* | (0.03) | 0.03 | (0.29) |
| Head of Household Education | |  |  |  |  |  |  |  |  |  |
| Primary or  higher school | 0.07 | (0.32) | -0.02 | (0.79) | -0.02 | (0.79) | 0.04 | (0.57) | -0.09 | (0.21) |
| Primary Income Source | |  |  |  |  |  |  |  |  |  |
| Agriculture &  off-farm | -0.30*** | (0.00) | -0.14* | (0.03) | -0.04 | (0.53) | -0.03 | (0.68) | 0.01 | (0.80) |
| Head of Household Sex | |  |  |  |  |  |  |  |  |  |
| Male | -0.06 | (0.59) | -0.19* | (0.03) | -0.24** | (0.00) | -0.14 | (0.21) | -0.12 | (0.18) |
| FMD Herd Status | |  |  |  |  |  |  |  |  |  |
| Yes, infection | 0.23*** | (0.00) | 0.05 | (0.47) | 0.13+ | (0.08) | 0.21** | (0.00) | 0.27*** | (0.00) |
| Country |  |  |  |  |  |  |  |  |  |  |
| Uganda | 0.07 | (0.36) | 0.11 | (0.16) | 0.19* | (0.02) | 0.14+ | (0.07) | -0.06 | (0.45) |
| Log-Likelihood | -140 | | -148 | | -146 | | -146 | | -130 | |

Note: Probit models. Quarantine impacts; 1=Somewhat/very important, 0=Not important. Reference categories: Highest level of education completed = No formal education. Primary Income Source = Livestock only or livestock and crops. Sex = Female. FMD herd status = No reported FMD in past year. Country = Tanzania. AME=average marginal effects; FMD= Foot-and-mouth disease. P value: +p<0.10, * p<0.05, ** p<0.01, ***p<0.001. Prob > Chi2 where the null hypothesis is the multivariate probit estimation.

**Table A2** Household socioeconomic characteristics and FMD incidence on the number of quarantine restrictions as important (n=231)

|  | AME | SE |
| --- | --- | --- |
| Herd size | 0.15 | (0.16) |
| Head of Household Education | |  |
| Primary or higher school | 0.05 | (0.84) |
| Head of Household Sex | |  |
| Male | -0.90+ | (0.06) |
| Primary Income Source | |  |
| Agriculture & off-farm | 0.06 | (0.88) |
| FMD Herd Status | |  |
| Yes, infection | 0.89*** | (0.00) |
| Country |  |  |
| Uganda | 0.41 | (0.13) |
| Log-likelihood | -414 | |
| Pearson $\chi^{2}$ = 182; df (224) = 0.81 | |  |

Note: Poisson model with no significant over/under dispersion (where deviance close to 1 suggests no over/under dispersion). Quarantine impacts; 1=Somewhat/very important, 0=Not important. Reference categories: Highest level of education completed = No formal education. Primary Income Source = Livestock only or livestock and crops. Sex = Female. FMD herd status = No reported FMD in past year. Country = Tanzania. AME=average marginal effects; FMD= Foot and mouth disease. P value: +p<0.10, * p<0.05, ** p<0.01, ***p<0.001.
